# Arsenic sulfide enhances the therapeutic effect of hepatocellular carcinoma immunotherapy through STAT3-THBS1/CD47 pathway

**DOI:** 10.3389/fimmu.2025.1612318

**Published:** 2025-09-11

**Authors:** Ting Kang, Zhuowei Feng, Yu Cai, Ruizhe Huang, Ruiheng Wang, Zhiyi Liu, Shumin Lu, Shufeng Xie, Han Liu, Siyu Chen

**Affiliations:** ^1^ Department of Oncology, Xin Hua Hospital, Shanghai Jiao Tong University School of Medicine, Shanghai, China; ^2^ Shanghai Institute of Hematology, State Key Laboratory of Medical Genomics, National Research Center for Translational Medicine at Shanghai, Ruijin Hospital, Shanghai Jiao Tong University School of Medicine and School of Life Sciences and Biotechnology, Shanghai, China

**Keywords:** arsenic sulfide, HCC, immunotherapy, THBS1, STAT3

## Abstract

**Background:**

Hepatocellular carcinoma (HCC) represents a formidable challenge in oncology, with high mortality rates and limited therapeutic options, particularly for advanced-stage patients. While immunotherapy has shown promise, its efficacy in advanced HCC remains suboptimal, necessitating the exploration of more potent therapeutic strategies.

**Methods:**

The HCC cell lines underwent treatment with arsenic sulfide and/or anti-PD1, while HepG2/Hepa1–6 cells were transduced with lentiviruses for THBS1 overexpression or knockdown. The MTT assay, FACS, Western blotting, qRT-PCR, and ChIP were employed to assess proliferation, modulation of proteins and genes. Additionally, C57BL/6J mice were utilized *in vivo* to investigate the ability of arsenic sulfide to enhance the efficacy of anti-PD-1 therapy.

**Results:**

Here, we investigated the role of arsenic sulfide in HCC treatment and explored its potential synergistic effects and underlying mechanisms when combined with immunotherapy. First of all, using bioinformatics analysis and validation *in vitro*, we identified thrombospondin-1 (THBS1) as a key prognostic factor for HCC in Asian populations. Then, we demonstrated that arsenic sulfide inhibits HCC cell viability, induces apoptosis, and downregulates THBS1 expression. Furthermore, we observed that arsenic sulfide significantly enhances the anti-HCC effects of anti-PD-1 therapy. Mechanistic insights indicate that arsenic sulfide inhibits STAT3 phosphorylation, reduces THBS1 transcription, thereby disrupting the binding between tumor cell THBS1 and T cell CD47, consequently enhancing anti-PD-1 efficacy. Therefore, arsenic sulfide augments anti-PD-1 efficacy against HCC by inhibiting the STAT3-THBS1/CD47 pathway.

**Conclusions:**

Collectively, our findings elucidate the role of arsenic sulfide in conjunction with PD - 1 in HCC eradication and its underlying molecular mechanism, providing a precise scientific rationale and a robust theoretical basis for arsenic sulfide’s application in HCC treatment.

## Introduction

Primary liver cancer ranks as the third most fatal malignancy worldwide, with hepatocellular carcinoma (HCC) representing 75%-85% of cases (1, 2). The advent of immunotherapy, notably immune checkpoint inhibitors (ICI) such as PD - 1/PD-L1 antibodies, has revolutionized cancer treatment by harnessing the power of the immune system to target cancer cells (3, 4). Immunotherapy has recently been established as the first-line treatment for advanced HCC. However, due to HCC’s heterogeneous and aggressive nature, anti-PD-1/PD-L1 as monotherapy achieves objective remission rates of only approximately 15%-20% in advanced HCC patients (5–8). While ICI combinations show potential to improve the clinical response, they achieve an improvement of objective response rate only up to approximately 30% (8, 9). The 5-year overall survival rate is still below 50%, the efficacy of immunotherapy is limited by frequent drug resistance (5, 7, 10), immunosuppressive tumor microenvironment (11), highlighting the need for more effective therapeutic strategies.

Arsenic compounds have shown cytotoxic activities in solid tumors. Currently, several studies have investigated the immunomodulatory effects of arsenic compounds. Arsenic trioxide (ATO) has emerged as a potent inducer of immunogenic cell death, and researchers have shown that combination of an ATO-based therapeutic vaccine and an immune checkpoint inhibitor against PD - 1 generated synergistic benefits against solid tumors (12). ATO delayed hepatic carcinoma growth, not only by depleting Treg but also *via* increased CD3 ^+^ T cells (13). Compared with ATO, arsenic sulfide (As_4_S_4_) has the advantages of being relatively safe, abundant, and orally administered. Recent studies have highlighted arsenic sulfide’s therapeutic potential in cancers such as gastric cancer (14), colon cancer (15), rhabdomyosarcoma (16) and osteosarcoma (17). Specifically, our group has reported that arsenic sulfide effectively suppresses HCC cell proliferation, induces apoptosis, and inhibits metastasis by targeting the HIF - 1α/VEGF pathway (18). However, the immunomodulatory effects of arsenic sulfide in solid tumors remain uncharacterized, and its potential as an immunotherapy adjuvant warrants further investigation.

Thrombospondin-1 (THBS1) is a multifunctional glycoprotein that belongs to the thrombospondin family, which is involved in a myriad of biological processes including cell adhesion, migration, proliferation, and communication by binding to receptors or proteins such as CD36, CD47, transforming growth factor beta, and integrins (19). The role of THBS1 in cancer is complex and appears to be highly dependent on the tumor type and stage (19). Elevated levels of THBS1 have been correlated with increased tumor aggressiveness and resistance to therapy (20, 21), making it a potential therapeutic target. Disruption of interactions between CD47 and THBS1 heightens cancer cell susceptibility to immune attack (22). Moreover, its interaction with immune cells and its role in modulating the tumor microenvironment highlight THBS1 as a key player in the complex interplay between cancer cells and the host immune system.

This study demonstrates that arsenic sulfide inhibits THBS1 expression by suppressing STAT3 phosphorylation. Consequently, this inhibition disrupts the interaction between THBS1 and CD47 in immune cells, modulates the body’s immune response, enhances the effectiveness of PD - 1 immunotherapy, and inhibits HCC progression. Our findings elucidate the role of arsenic sulfide in combination with anti-PD-1 therapy in HCC eradication and its underlying molecular mechanism, providing a precise scientific rationale and a robust theoretical basis for arsenic sulfide’s application in HCC treatment.

## Materials and methods

### Cell culture and reagent

HepG2 and Hepa1–6 were purchased from the Cell Bank, Chinese Academy of Sciences (Shanghai, People’s Republic of China). Experiments were performed on cell lines passaged fewer than 30 times. Mycoplasma was tested monthly following an established procedure (14). Cells were cultured in DMEM containing 10% FBS and 100 U/mL penicillin–streptomycin at 37 °C. Cell viability was analyzed using the CCK8 (TargetMol, Catalog No. C0005). Highly purified arsenic sulfide was supplied by the Shanghai Institute of Hematology (Shanghai, People’s Republic of China) and was prepared as previously described (23). Anti-PD-1 antibodies such as Pembrolizumab (Catalog #A2005), Nivolumab (Catalog #A2002), Tislelizumab (Catalog #A3039), Camrelizumab (Catalog #A2016), rat IgG2a isotype control (clone 2A3), and anti-mouse PD - 1 (CD279) (clone RMP1 - 14) were also sourced from Selleckchem.

### Flow cytometry

The apoptosis was performed using the Annexin V-FITC Apoptosis Detection Kit (BD Pharmingen). Human CD69-PerCP-Cy5.5 and LAG3-PE antibodies were obtained from BD Biosciences. Flow cytometry data were analyzed using the FlowJo software.

### Immunoblot

Protein lysates, loaded at equal quantity, were separated on a NuPAGE Novex 10% Bis-Tris Protein Gel (Thermo Fisher Scientific), followed by iBlot transfer to PVDF (Thermo Fisher Scientific). Membranes were blocked in 5% skim milk powder in PBS containing 0.1% Tween-20, and then probed with the following antibodies, as indicated in the figure legends: THBS1(ab267388, Abcam), S100P(ab124743, Abcam), FCN2(ab267473, Abcam), CD47 (Cat No:66304-1-Ig, Proteintech), β-actin (A1978, Sigma-Aldrich), STAT3(A1192, ABclonal), p-STAT3(Y705)(AP0705, ABclonal), PD-L1(A1645, ABclonal) followed by goat anti–rabbit IgG antibodies conjugated to HRP or goat anti–mouse IgG antibodies conjugated to HRP, respectively (Southern Biotech). Immunoblot signals were acquired with the Amersham Imager 600 (General Electric Company, Boston, MA, USA).

### Quantitative reverse-transcription PCR

RNA was isolated with Spin Column Animal Total RNA Purification Kit (Sangon Biotech) and reverse-transcribed with the High-Capacity cDNA Reverse Transcription Kit (Thermo Fisher Scientific). cDNA was amplified using SYBR-Green PCR Master Mix (Thermo Fisher Scientific). When possible, primers were designed to span exon-exon junctions. Gene expression fold changes were normalized to GAPDH. Primer sequences are listed in **Supplementary Table S1**.

### Lentiviral vectors

The lentiviral vector (Ubi-MCS-3FLAG-CMV-EGFP) for human THBS1 overexpression was purchased from GeneChem (Shanghai, China). Target sequence (GTAGGTTATGATGAGTTTAAT and CGTGACTGTAAGATTGTAAAT) against THBS1 were inserted into the pLKO.1-TRC vector, according to the manufacturer’s protocol (Addgene). Lentiviral particles were produced by calcium phosphate transfection into HEK - 293T cells with helper plasmids. Supernatants were collected, filtered, and used for transduction with polybrene (Sigma-Aldrich). Transduced cells were FACS-purified based on the fluorescent reporter protein.

### Wound healing assays

Monolayers of cells were plated in 24-well plates and then scratched with the tip of a 20μL pipette and washed several times with PBS (Hyclone) until dislodged cells removed clearly. Tumor cells were cultured in DMEM medium free from FBS. The wound area was photographed at 0h and 48h post-scratching.

### Chromatin immunoprecipitation

Cells (1 × 10^7) were used per ChIP assay according to a published protocol (24). Briefly, cells were crosslinked with 1% paraformaldehyde for 15 min and were quenched with glycine for 5 min at room temperature. Fixed chromatin was digestion with micrococcal nuclease and immunoprecipitated with the p-STAT3(Y705) (AP0705, ABclonal) antibody. ChIP-qPCR was performed using SYBR-Green PCR Master Mix (Thermo Fisher Scientific) on a ViiA7 PCR machine (Applied Biosystems). Relative enrichments are presented as percentage input. Primer sequences are available on **Supplementary Table S1**.

### Immunofluorescence

Before co-culture, CD3^+^T Cells were labeled with celltrace violet (CellTrace Violet Proliferation Kit, Invitrogen). Cytospin preparations of collected supernatant cells were air-dry and fixed by 4% paraformaldehyde at room temperature for 15 minutes, blocked with 1% BSA for 1h, and then stained using primary antibody mouse-anti CD47. The secondary antibodies used were anti-mouse Alexa Fluor 594 dye conjugate (Life Technologies). Finally, the slides were mounted and imaged under Zeiss LSM 800 confocal microscope.

### Multiplex cytokine profiling

Cytokines were analyzed by LEGENDplex bead-based immunoassays (BioLegend, San Diego, USA) according to the manufacturer’s instructions. The human CD8/NK panel (740267, Biolegend) were used to simultaneously quantify 13 serum cytokines/chemokines, including IL - 2, 4, 6, 10, 17A, IFNγ, TNFα, soluble Fas (sFas), soluble FasL (sFasL), granzyme A, granzyme B, perforin and granulysin. Data acquisition was performed on a BD FACS Calibur flow cytometer (BD Biosciences) and analyzed with the LEGENDplex™ Data Analysis Software (BioLegend).

### Subcutaneous models

All C57BL/6 mice (4- to 6-week-old male) were obtained from Shanghai Slac Laboratory Animal Co. and maintained in a 12 hours light/dark cycle with free access to food and water. To establish the subcutaneous HCC model, Hepa1–6 cells (5 × 10^6^ cells in 100 μL of phosphate buffer saline [PBS]) were injected into the right flanks of mice. A total of n=20 animals when tumors reached ~50 mm^3 were randomized using the random numbers generator into four groups (n=5) and treated as follows: vehicle+IgG (10 mg/kg), arsenic sulfide (2 mg/kg), anti-PD-1 (10 mg/kg), or combination therapy. Anti-PD-1 or control IgG were injected intraperitoneally three times per week, and arsenic sulfide was injected every other day for 2 weeks. Tumor volumes were measured every three days with calipers, calculated using the formula a × b^2/2 (where a and b are the largest and smallest tumor diameters), and analyzed. The animals were euthanized following anesthesia when the tumor volume exceeded 1500 mm^3^. Tumor tissue was removed following the final treatment. All animal procedures were conducted according to protocols approved by the Institutional Animal Care and Ethics Committee of Shanghai Jiao Tong University.

### Peripheral blood or tumor infiltrating lymphocyte analysis

Mouse PBMC and Purified tumor‐infiltrated lymphocyte were resuspended in 2% fetal bovine serum (FBS)/PBS buffer and then filtered through 40-μm cell strainers. For surface antigens, such as live/died (Fixable Viability Stain 510, 564406), CD45(PerCP-Cy5.5 Rat Anti-Mouse CD45(30-F11), 550994), CD3(FITC Hamster Anti-Mouse CD3e(145 - 2C11), 553061), CD4(PE Rat Anti-Mouse CD4(RM4 - 5), 553048), CD8(APC Rat Anti-Mouse CD8a(53 - 6.7), 553035) from BD Pharmingen and PD‐1(Anti-Mouse PE-Cy7(J43), 25 - 9985-82) from Invitrogen, single cells were stained with corresponding antibodies and incubated in the dark on ice for 20 min. Cells were washed twice and resuspended in 2% FBS or PBS, then detected by flow cytometry. Data analysis was performed using FlowJo software.

### Immunohistochemistry staining

Tumors were embedded in paraffin, and CD8 or CD4 staining were performed using the anti-CD8 antibody (clone 4SM15, 14 - 0808-82, 1/500, Invitrogen), the anti-CD4 antibody (clone 4SM95, 14 - 9766-82, 1/1000, Invitrogen), DAB (brown) and counterstaining with hematoxylin. Images were digitalized with a 3DHistech Pannoramic SCAN2 scanner. Quantification of the CD4/CD8 area was performed with Image J.

### T cell cytotoxic assay

For the T cell cytotoxic assay: CD3^+^T(using EasySep Human T Cell Isolation Kit), CD4^+^T(MojoSort™ Human CD4 T Cell Isolation Kit, Cat #480010, Biolegend) and CD8^+^T(MojoSort™ Human CD8 T Cell Isolation Kit, Cat# 480012, Biolegend) cells isolated from healthy individuals peripheral blood mononuclear cell (PBMC) were pre-activated by human T-activator CD3/CD28 (Miltenyi Biotec) in a 12-well plate at a concentration of 10^5^ cells/well, culturing for 72h under 37 °C.

On Day 1, HCC cells were seeded into 24-well plates. On Day 2, activated CD3^+^, CD4^+^, or CD8^+^ T cells were co-cultured with HCC cells at a ratio of 5:1 or 1:1 in DMEM (Hyclone) supplemented with 10% FBS (Gibco), in the presence or absence of anti-PD-1 antibodies and arsenic sulfide. Co-culture was maintained at 37 °C in a humidified incubator with 5% CO_2_ for 48 hours.

After the co-culture, the supernatant as well as the floating CD3^+^T, CD4^+^T or CD8^+^T cells were removed. Remaining HCC cell colonies were fixed in 4% formalin for 20 minutes and stained with 1% crystal violet for 20 minutes. Colony numbers were counted to assess the cytotoxic activity of CD3^+^, CD4^+^ or CD8^+^ T cells. Each experiment was repeated for three times.

### Statistical analysis

All statistical analyses were conducted using the GraphPad Prism package (PRISM 9.1.0; GraphPad Software). The results of this study are presented as mean ± SEM and are derived from at least three independent experiments. One-way or two-way ANOVA was applied for multiple comparisons, while Student’s t-test was used for single comparisons. A *p* value of less than 0.05 was considered to be statistically significant. Statistical significance is denoted as follows: *P < 0.05, **P < 0.01, ***P < 0.001, ****P < 0.0001; ns, no significance.

## Results

### THBS1 emerges as a prognostic factor for hepatocellular carcinoma in Asian populations

**Figure 1:**
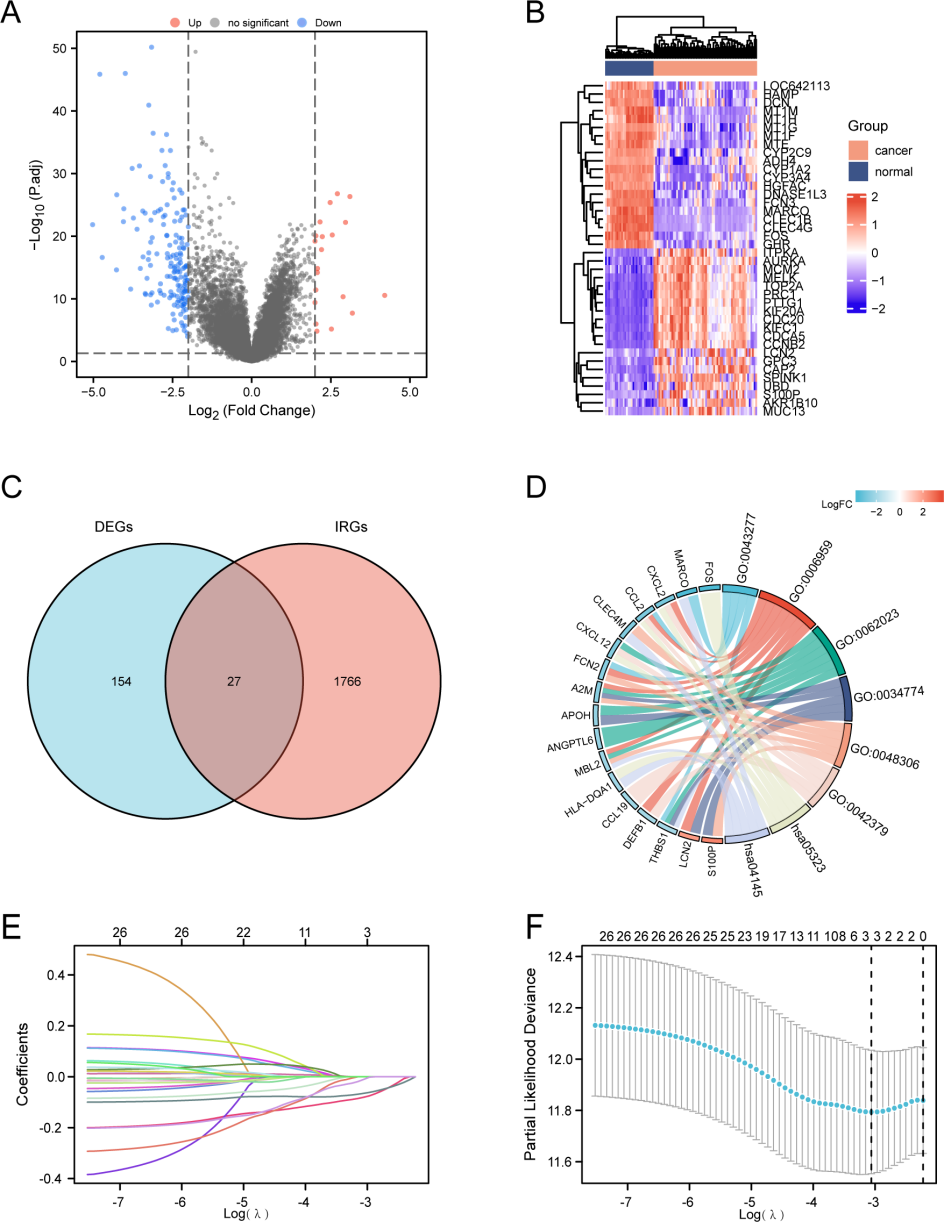
Identification of prognostic differentially expressed immune-related genes (DEIRGs) in HCC. **(A)** Volcano plot of significantly differentially-expressed genes (DEGs) between HCC and normal tissues of the GSE87630 dataset. |log2 (Fold Change) |>2 was considered as significant. **(B)** Heatmap showing the top 20 genes with the most pronounced upregulation and downregulation in HCC compared to normal tissues. **(C)** Venn diagram visualizing the intersections of DEGs (n=181) with immune-related genes (IRGs) from ImmPort (n=1791). Genes included by both groups were considered as DEIRGs (n=27). **(D)** GO and KEGG enrichment chord diagram indicating the interactions among DEIRGs. **(E, F)** LASSO Cox regression with a 10-fold cross-validation for the prognostic value of the DEIRGs. TCGA-LIHC dataset was used as the training set. Univariate Cox analysis of overall survival (OS) was performed to screen DEIRGs with prognostic values, and the optimal λ values were extracted to eliminate the false-positive ones.

Although immunotherapy has become a first-line treatment for advanced HCC, predictive biomarkers to identify patients who will benefit most from immunotherapy remain absent. To address this question, we analyzed differentially expressed genes (DEGs) from the GEO dataset (GSE87630), identifying 181 DEGs with a false discovery rate (FDR) < 0.05 (**Figure 1A**). Among these, 20 genes exhibited upregulation, while 161 showed downregulation. The heatmap plots highlight the top 20 upregulated and downregulated genes (**Figure 1B**). By comparing these DEGs with the 1793 immune-related genes (IRGs) sourced from the ImmPort database, we discovered 27 overlapping IRDEGs (**Figure 1C**). Subsequently, these IRDEGs were subjected to Gene Ontology (GO) and Kyoto Encyclopedia of Genes and Genomes (KEGG) pathway analyses, with the associated functional pathways visualized in a chord plot format (**Figure 1D**). Furthermore, a LASSO-Cox regression analysis was applied to the IRDEGs, facilitating the creation of a predictive risk score for overall survival. The coefficients for each IRDEG are detailed in **Figure 1E**, while **Figure 1F** illustrates the risk model developed from the 27 selected IRDEGs. Finally, three prognostic related IRDEGs, there are THBS1, FCN2 and S100P, were selected. Together, these data indicate that THBS1, FCN2 and S100P may emerged as prognostic factors for HCC in Asian populations.

### Arsenic sulfide inhibits HCC viability, promotes apoptosis, and suppresses THBS1 expression

**Figure 2:**
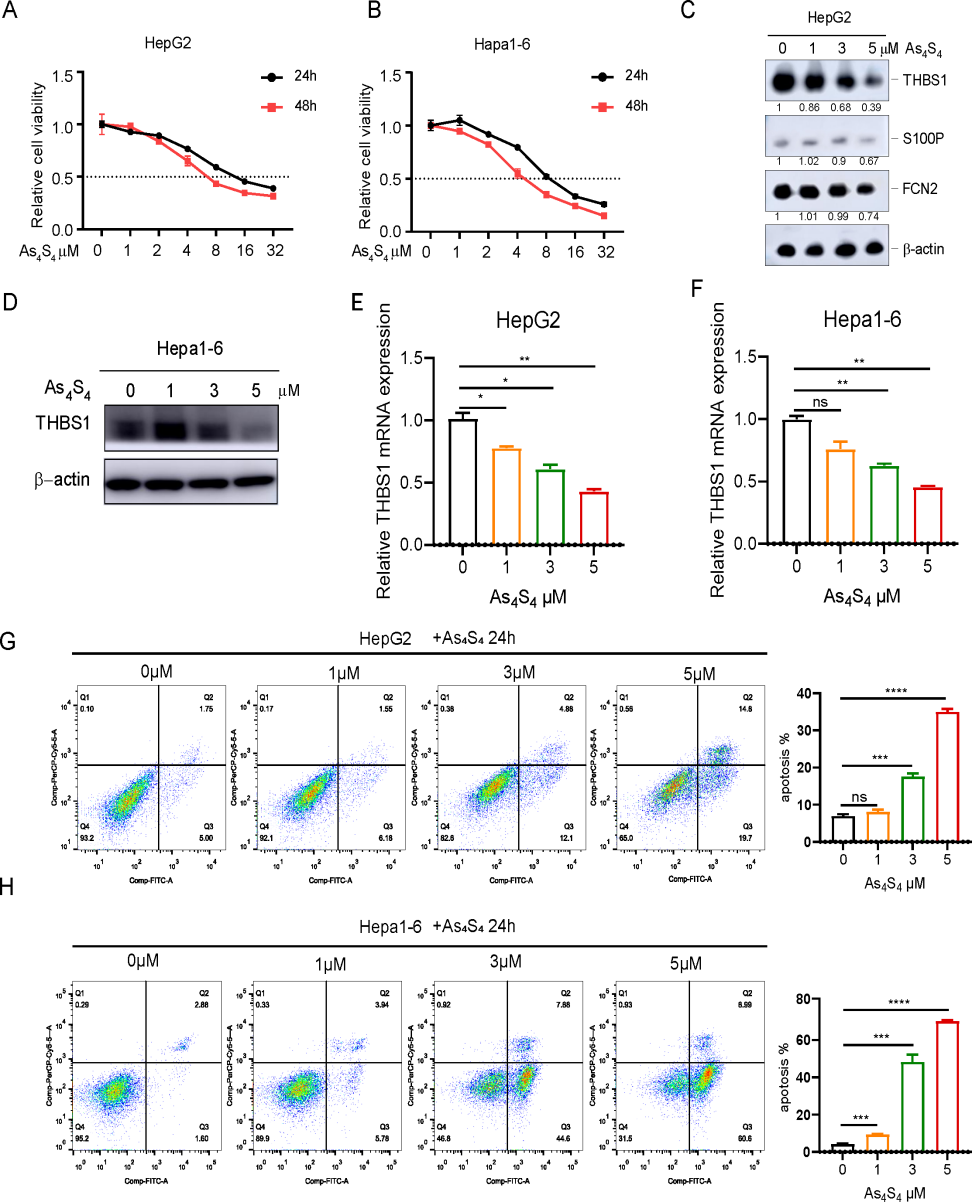
Arsenic sulfide inhibits viability of HCC, promotes apoptosis, and suppresses the expression of THBS1. **(A, B)** Cell proliferation assays showing the effect of arsenic sulfide on the growth of HepG2 (human) and Hepa1-6 (mouse) HCC cell lines. **(C)** Immunoblot analysis of THBS1, S100P and FCN2 expression in arsenic sulfide treated HepG2 cells. **(D)** Immunoblot analysis of THBS1 expression in arsenic sulfide treated Hepa1–6 cells. **(E, F)** RT-PCR analysis of THBS1 mRNA expression in arsenic sulfide-treated HepG2 **(E)** and Hepa1-6 **(F)** cells. **(G, H)** Flow cytometry analysis quantifying apoptosis in HepG2 **(G)** and Hepa1-6 **(H)** cells exposed to different concentrations of arsenic sulfide. (Fold changes relative to untreated controls are indicated. (Data are shown as the mean ± SEM, *n* = 3. *P < 0.05, **P < 0.01, ***P < 0.001, ****P <0.0001; ns, no s significance).

Our previous study demonstrated that arsenic sulfide inhibits HCC cell proliferation, induces apoptosis, and blocks metastasis via the HIF - 1α/VEGF pathway (18). To gain additional insights, we further explored the mechanics of arsenic sulfide to inhibit HCC by focusing on three specific IRDEGs. Both the human HCC cell lines HepG2 (**Figure 2A**), Hep3B (**Supplementary Figure S1A**) and the mouse HCC cell line Hepa1-6 (**Figure 2B**), were exposed to a range of arsenic sulfide concentrations, which led to a significant suppression of cell proliferation. After 24-hour treatment, THBS1 protein levels progressively decreased in HepG2 cells, while no significant changes were observed in FCN2 and S100P (**Figure 2C**). A comparable reduction in THBS1 protein level was noted in Hep3B (**Supplementary Figure S1B**) and the mouse Hepa1–6 cells post-treatment with arsenic sulfide (**Figure 2D**), along with a gradual decline in THBS1 mRNA level in both HepG2 and Hepa1–6 cells as the arsenic sulfide concentration increased (**Figures 2E, F**). Conversely, arsenic sulfide had minimal effects on FCN2 and S100P, reinforcing a specific association between arsenic sulfide and THBS1 suppression in HCC treatment. We also found that THBS1 protein levels progressively decreased in HepG2 and Hep3B cells after 3μM arsenic sulfide treatment in different time (**Supplementary Figure S1C**). In addition, arsenic sulfide markedly induced apoptosis in the HCC cells in a dose-dependent and time-dependent manner (**Figure 2G, H**, **Supplementary Figure S1D-F**). Collectively, these findings indicate that arsenic sulfide inhibits HCC cell viability, promotes apoptosis, and suppresses THBS1 expression.

### Arsenic sulfide suppresses THBS1 expression by inhibiting STAT3 phosphorylation and transcriptional activity

**Figure 3:**
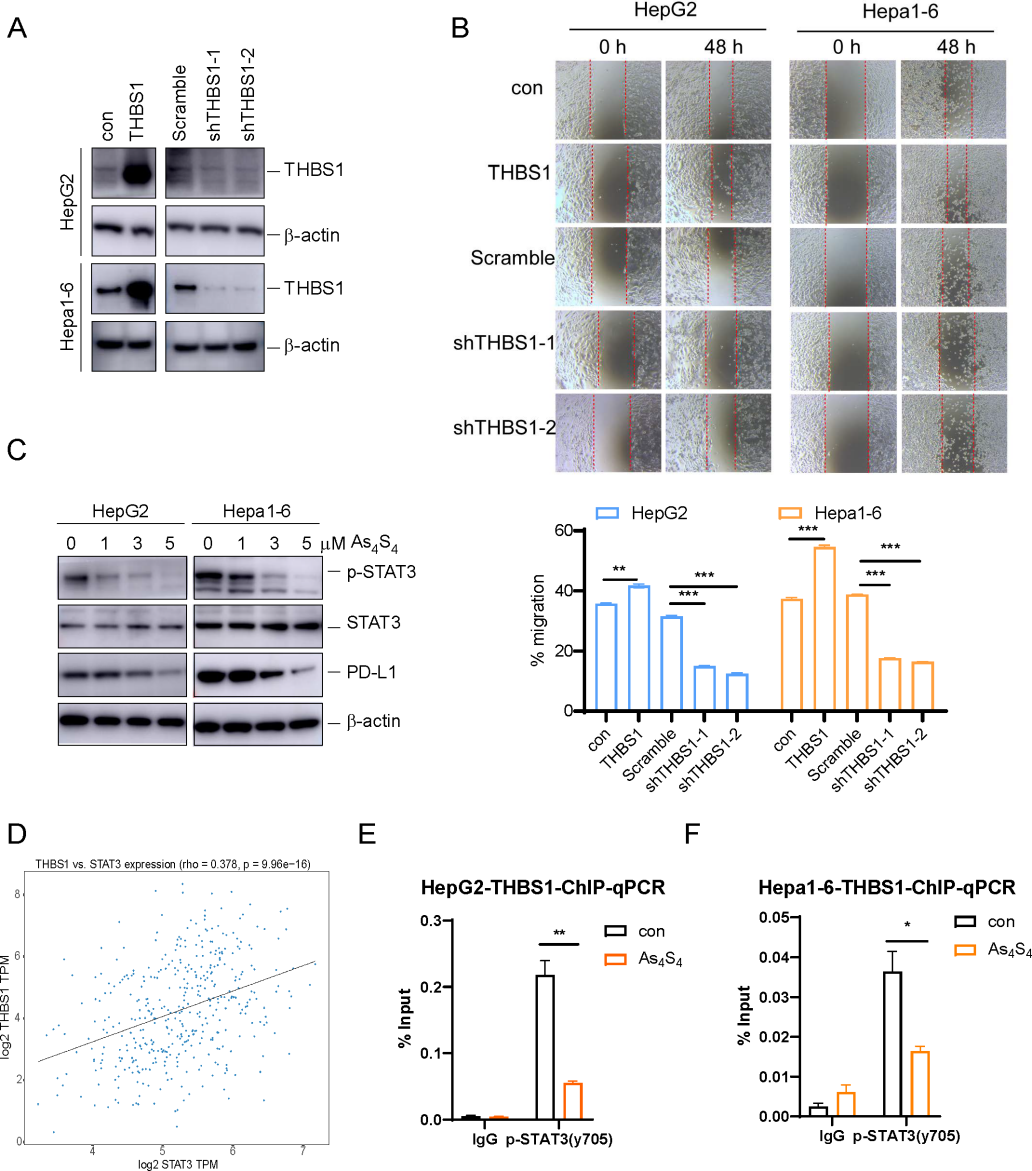
Arsenic sulfide suppresses THBS1 expression by inhibiting STAT3 phosphorylation and transcriptional activity. **(A)** Immunoblot validation of THBS1 interference and overexpression regulation in HepG2 and Hepa1–6 cells. **(B)** Cell scratch assay in THBS1 expression-regulated cells (THBS1 overexpression, scramble, shTHBS1-1, shTHBS1-2) compared to control cells. Cell migration capacity was analyzed by scratch width after 48 h. **(C)** Immunoblot analysis of p-STAT3, STAT3 and PD-L1 expression in arsenic sulfide treated HepG2 and Hepa1–6 cells. **(D)** Plot of the correlation between the expression of THBS1 and STAT3 based on The Cancer Genome Atlas data. **(E, F)** ChIP-PCR analysis of promoter regions with antibodies targeting p-STAT3 in arsenic sulfide treated HepG2 **(E)** and Hepa1–6 cells **(F)**. (Data are shown as the mean ± SEM, *n* = 3. *P < 0.05, **P < 0.01, ***P < 0.001; ns, no significance).

To understand the functions of THBS1 in HCC, lentiviral vectors were employed to either overexpress or silence THBS1 in HepG2 and Hepa1–6 cell lines. The success of these genetic manipulations was validated through Western blot analysis (**Figure 3A**). Additionally, scratch wound healing assays demonstrated that overexpression of THBS1 significantly enhanced the migratory capacity of tumor cells, whereas its suppression had the converse effect, impeding cell migration (**Figure 3B**). Previous studies have shown that p-STAT3 can regulate THBS1 expression at the transcriptional level (25). As mentioned above, arsenic sulfide could inhibit the expression of THBS1, possibly through transcriptional regulation by p-STAT3. To validate this hypothesis, we evaluated the expression patterns of p-STAT3 in both the HepG2 and Hepa1–6 cell lines after arsenic sulfide treatment. Our findings revealed that arsenic sulfide treatment attenuated STAT3 phosphorylation (**Figure 3C** and **Supplementary Figure S2A**) and reduced the expression of programmed death-ligand 1 (PD-L1) (**Figure 3C**). A positive correlation between STAT3 and THBS1 expression was observed in gene expression profiles of TCGA (**Figure 3D**). To investigate the potential role of STAT3-THBS1 pathway, we calculated its activation score using ssGSEA based on RNA-seq data from the TCGA-LIHC cohort. By comparing activation scores across different immunophenoscore (IPS) groups, we found that patients predicted to be more responsive to immune checkpoint inhibitors tended to exhibit lower STAT3-THBS1 pathway activation levels (**Supplementary Figure S2B**). Survival analysis also showed a correlation between the pathway activation and overall survival of HCC patients (**Supplementary Figure S2C**). Additionally, the STAT3-THBS1 pathway activation score was significantly positively correlated with the expression of key immune checkpoint genes, including PD - 1 and CTLA - 4 (**Supplementary Figure S2D**). These results suggest that inhibition of the STAT3-THBS1 pathway may improve responsiveness to immunotherapy. Chromatin immunoprecipitation (ChIP) assays confirmed the binding of p-STAT3 (Tyr705) to the promoter region of the THBS1 gene. These regions contain putative p-STAT3 binding sites (**Supplementary Figure S2E**), suggesting that THBS1 expression is directly regulated by p-STAT3 (**Figure 3E, F**). In addition, IGV-based visualization revealed a prominent STAT3 binding peak at the THBS1 promoter region, providing further evidence that STAT3 may transcriptionally regulate THBS1 expression (**Supplementary Figure S2F**). Concurrently, we noted that THBS1 levels decreased upon arsenic sulfide treatment (**Figures 2C, D**), and this decrease was also associated with decreased p-STAT3 occupancy at the THBS1 promoter (**Figures 3E, F**). These data suggested that one function of arsenic sulfide is to continuously repress expression of THBS1 by inhibiting STAT3 phosphorylation and transcriptional activity, thereby limiting HCC proliferation.

### THBS1 inhibits T cell-mediated killing of HCC via CD47, and arsenic sulfide counteracts this effect

**Figure 4:**
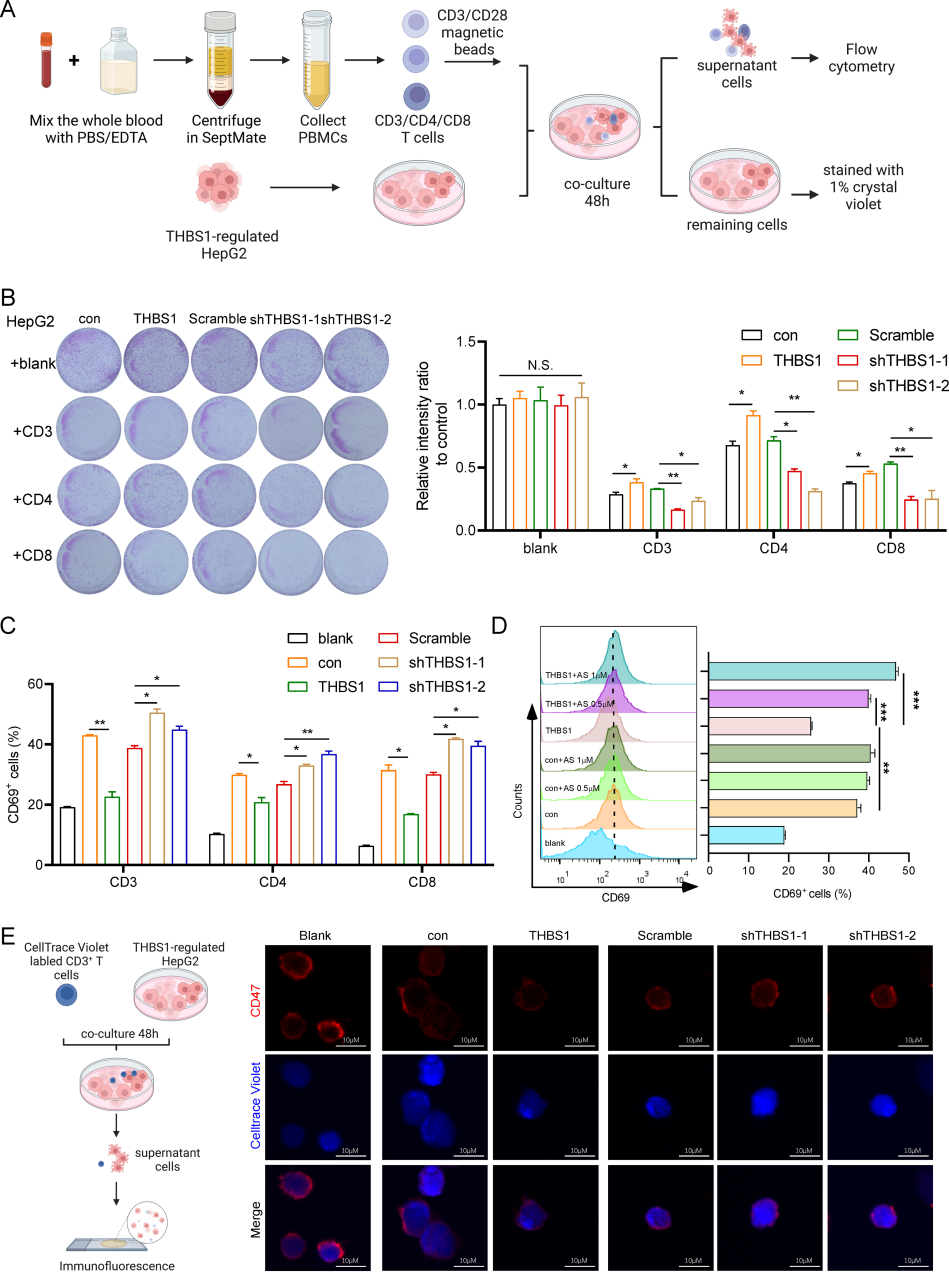
THBS1 inhibits T cells from killing HCC via CD47 and arsenic sulfide counteracts this phenomenon. **(A)** Schematic of T cell cytotoxicity assay in THBS1 expression-regulated HepG2 cells. CD3^+^, CD4^+^, CD8^+^T cells were isolated from human PBMC, pre-activated with CD3/CD28 magnetic beads and co-cultured for 48h with THBS1-regulated HepG2 cells (con, THBS1, scramble, shTHBS1-1, shTHBS1-2). The remaining tumor cells were quantified by relative intensity after staining. **(B)** CD3^+^, CD4^+^, CD8^+^T cell cytotoxicity assay was performed to evaluate the killing capability of T cells in THBS1-regulated HepG2 cells. **(C)** Flow cytometry analysis of CD3^+^, CD4^+^, CD8^+^T cell activation levels following cytotoxicity assay. **(D)** Flow cytometry analysis of activation levels of CD3^+^T cells co-cultured with THBS1 overexpressing cells, with or without arsenic sulfide treatment. **(E)** Immunofluorescent staining of T cells to identify CD47 changes after coculture with THBS1-regulated HepG2 cells. (Data are shown as the mean ± SEM, *n* = 3. *P < 0.05, **P < 0.01, ***P < 0.001; ns, no significance).

Given that cancer immunotherapy depends mainly on T-cell infiltration in the tumor immune microenvironment (11), we isolated CD3^+^ T cells, CD4^+^ T cells and CD8^+^ T cells from healthy human peripheral blood mononuclear cells (PBMCs) to performed a cytotoxicity assay (**Figure 4A**). Subsequently, isolated T cells were activated with CD3/CD28 magnetic beads. We then established an *in vitro* coculture system with isolated T cells and THBS1-regulated HepG2(con, THBS1, Scramble, sh-THBS1-1, sh-THBS1-2) for 48 hours (**Figure 4A**). Among the T cell subsets, CD3^+^ T cells exhibited the highest cytotoxic activity against HepG2 cells, followed by CD8^+^ T cells (**Figure 4B**). Notably, overexpression of THBS1 reduced T cell-mediated cytotoxicity, while THBS1 knockdown enhanced it (**Figure 4B**). Additionally, analysis of T cell activation marker CD69 revealed a corresponding trend between T cell activation and killing efficacy (**Figure 4C**). Specifically, THBS1 overexpression dampened T cell activation, particularly CD3^+^T cells activation, whereas THBS1 interference significantly enhanced T cell activation compared to scramble cells (**Figure 4C**), suggesting that THBS1 may inhibits immune cell-mediated killing. Since we found that arsenic sulfide can down-regulate THBS1 in HCC cell lines (**Figures 2C, D**), and THBS1 suppresses immune cell-mediated killing (**Figures 4B, C**), we hypothesized that arsenic sulfide treatment might counteract the suppressive effect of THBS1 on immune cells. To test this hypothesis, we co-cultured CD3^+^ T cells with HepG2-THBS1 cells and treated them with low concentrations of arsenic sulfide (0.5 or 1 μM). Results showed that arsenic sulfide restored T cell activation, with activation levels increasing with arsenic sulfide concentration (from 25.6% to 39.95% at 0.5 μM and 46.8% at 1 μM) (**Figure 4D**).

Several studies have reported that THBS1 interaction with CD47 on NK cells and cytotoxic T cells inhibits their activation and limits granzyme B production that mediates antigen-dependent lysis of tumor cells (26–28). We further investigated CD47 expression in T cells via immunofluorescent staining after co-culture with THBS1-regulated HepG2 cells (**Figure 4E**). As indicated, there was a marked decrease in CD47 fluorescence intensity when T cells coculture with HepG2-THBS1 compared to the control group (coculture with HepG2-con) (**Figure 4E**). Conversely, the fluorescence intensity of CD47 was significantly increased when THBS1 was knocked down in HepG2. Studies had demonstrated that CD47 knockout T cells were sensitive to macrophage-mediated phagocytosis (29–31). Combined with our findings, the THBS1-CD47 interaction played an impairment role in T cells killing HepG2 cells. Together, these data demonstrate that THBS1 inhibits T cell-mediated killing of HCC cells via CD47, and arsenic sulfide counteracts this immunosuppressive effect.

### Enhanced therapeutic efficacy of combination therapy with PD - 1 blockade and arsenic sulfide *in vitro*

**Figure 5:**
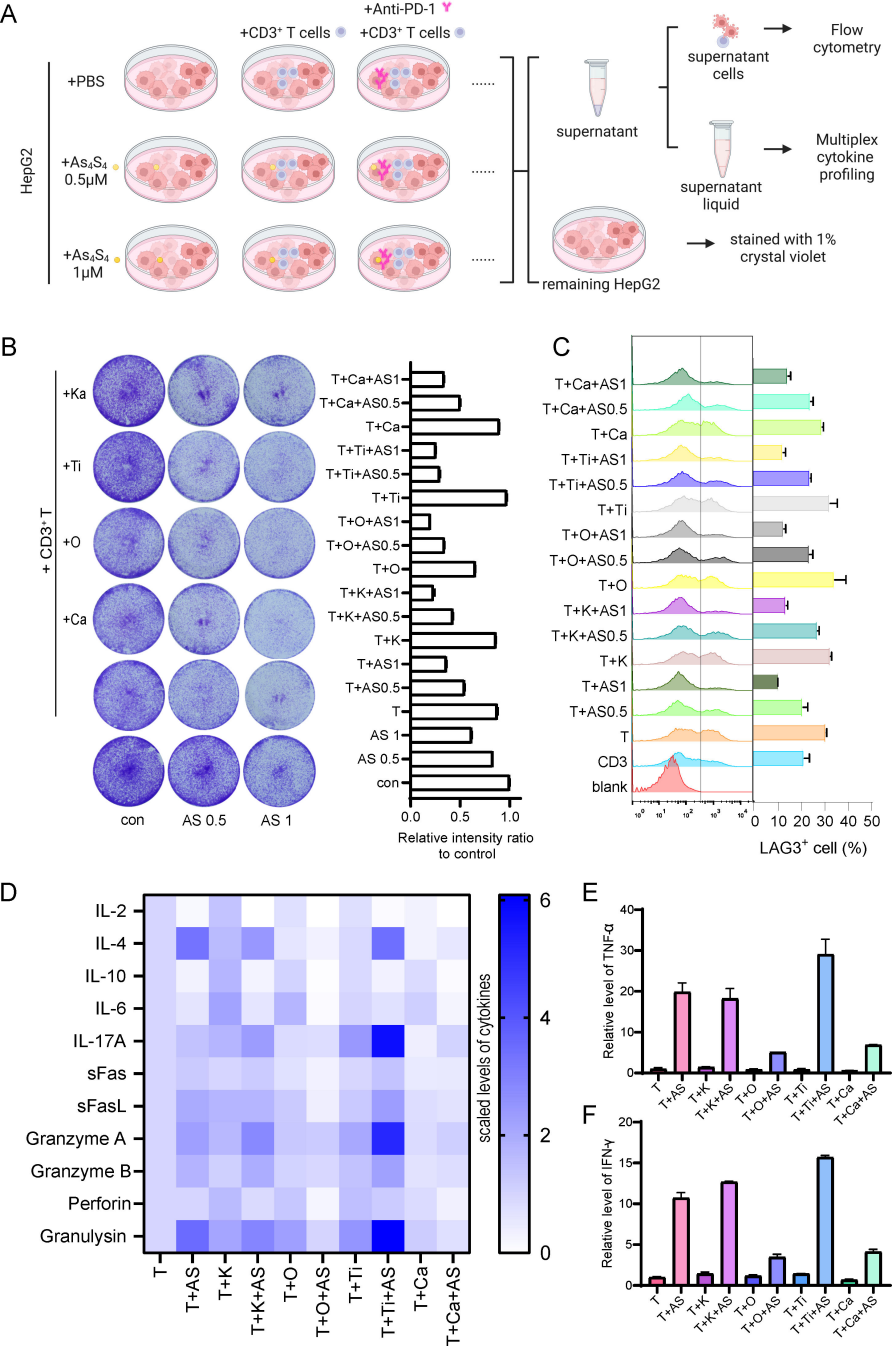
Combination Therapy with PD - 1 Blockade and Arsenic Sulfide *in vitro* **(A)** Schematic illustrating the experimental setup to evaluate the effects of various PD - 1 antibodies—Pembrolizumab (K), Nivolumab (O), Tislelizumab (Ti), and Camrelizumab (Ca)—in combination with arsenic sulfide and CD3^+^ T cells on HepG2 cells. CD3^+^T cells were isolated from human PBMC, pre-activated with CD3/CD28 magnetic beads and co-cultured for 48h with HepG2 cell with or without arsenic sulfide. **(B)** Quantification of the remaining tumor cells by relative intensity after staining. **(C)** Flow cytometry analysis assessing T cell exhaustion by measuring the expression of LAG - 3. **(D-F)** Flow cytometry analysis of multiplex cytokine profiling to detect secretion of cytokines [TNF-α **(E)** and IFN-γ **(F)**] by T cells after treated with different drugs. (Data are shown as the mean ± SEM*, n* = 3.).

Arsenic sulfide has the potential to modulate immune responses by suppressing THBS1 in tumor cells. The question arises whether its combination with immunosuppressive agents could yield enhanced therapeutic outcomes. To address this, we conducted experiments using various PD - 1 antibodies—Pembrolizumab (K), Nivolumab (O), Tislelizumab (Ti), and Camrelizumab (Ca)—in conjunction with arsenic sulfide and CD3^+^ T cells to treat HepG2 cells (**Figure 5A**). We assessed the inhibitory effects on tumor cells and analyzed T cell activation and exhaustion indicators. Our findings indicated that the combination of T cells and PD - 1 antibodies did not significantly differ in terms of tumor survival when compared to T cell therapy alone (**Figure 5B**). However, the integration of T cell treatment with arsenic sulfide led to an additional suppression of tumor cells (**Figure 5B**). Utilizing flow cytometry, we further analyzed T cell activation marker CD69, and exhaustion marker LAG3. The results demonstrated that all PD - 1 antibodies increased T cell activation, with nivolumab showing the most pronounced effect (the percentage of CD69^+^ T cells increased from 39.85% to 44.80%) (**Supplementary Figure S3A**). Further analysis of T cell exhaustion signals showed that arsenic sulfide treatment significantly reduced the expression of exhaustion signals (**Figure 5C**). Specifically, co-culturing T cells with tumor cells alone resulted in 30% of T cells expressing LAG3 (**Figure 5C**). This proportion was reduced to 21% and 10% following treatment with 0.5 µM and 1 µM arsenic sulfide, respectively. The addition of PD - 1 antibodies slightly elevated the level of T cell exhaustion. Next, to further investigate the underlying mechanism, cytokine profiling was detected. There was a general increase in cytokines in the presence of arsenic sulfide (**Supplementary Figure S3B**). We observed a significant increase in inflammatory factors, including IL - 4, IL - 17A, Granzyme A, Granzyme B, and Granulysin (**Figure 5D**), with the most notable upregulation observed in TNF-α (**Figure 5E**) and IFN-γ (**Figure 5F**). Thus, in the context of combination treatment, arsenic sulfide inhibits THBS1 in tumor cells while upregulating CD69 and downregulating LAG3 in T cells, it is reasonable to surmise that arsenic sulfide modulates T cell activity to augment the efficacy of anti-PD-1 therapy.

### Arsenic sulfide enhances the efficacy of PD - 1 blockade against HCC *in vivo*

**Figure 6:**
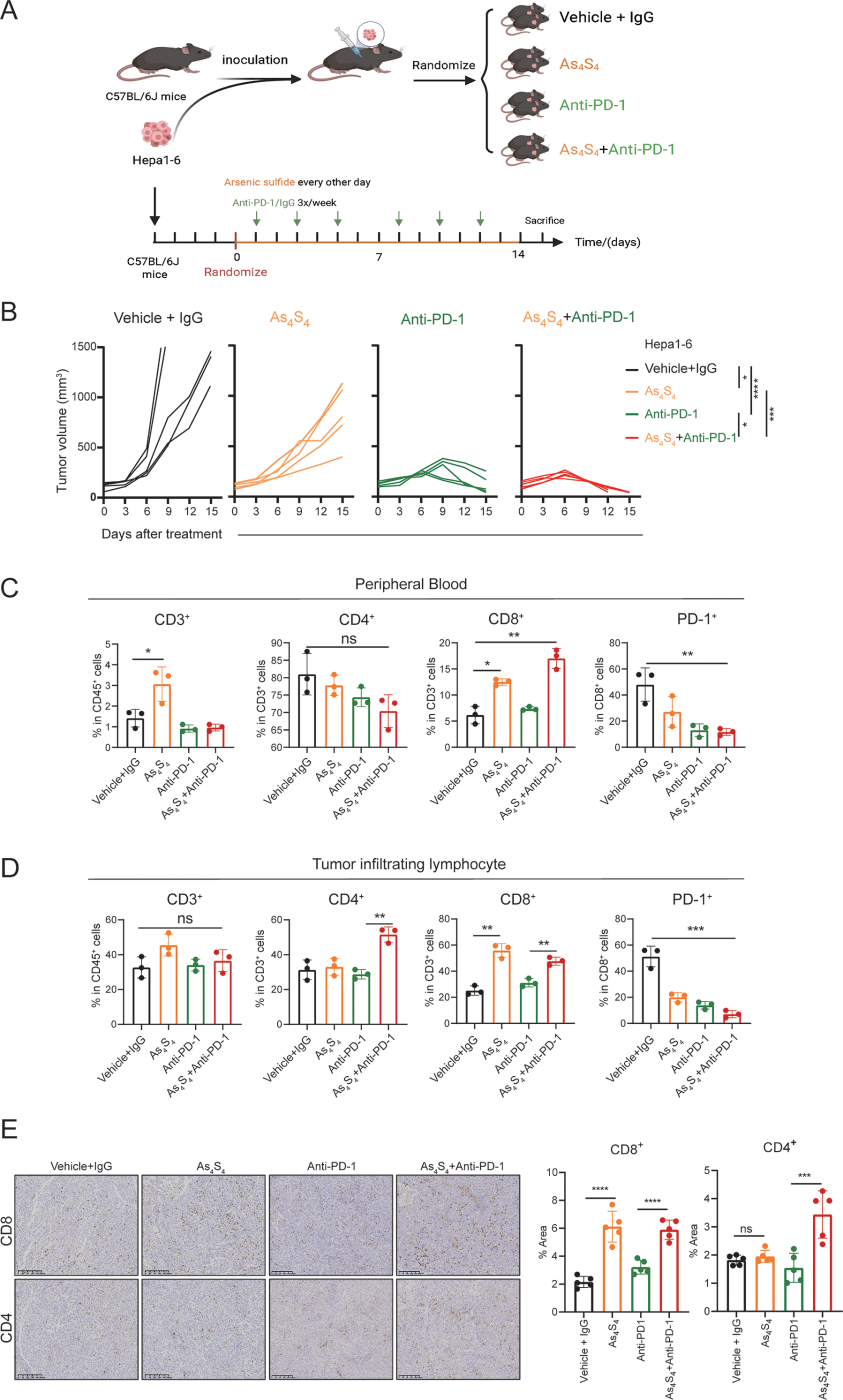
Arsenic Sulfide enhances the response of HCC to PD - 1 blockade *In Vivo*. **(A)** Schematic representation of the therapy schedule for arsenic sulfide, anti-PD-1 and combination therapy. Hepa1–6 cells were subcutaneously injected into C57BL/6 mice. When tumor volume reached 80 mm3, mice were randomized into four groups (n=5) and received indicated treatment. (vehicle+IgG, 10 mg/kg; As4S4, 2mg/kg; anti-PD-1, 10 mg/kg; combination therapy). **(B)** Tumor growth curves of each group. **(C, D)** Flow cytometry analysis of proportion of CD3^+^, CD4^+^, CD8^+^T cells and PD - 1 positivity of CD8^+^T cells in peripheral blood **(C)** and tumor-infiltrating lymphocytes **(D)** of mice after 2-weeks treatment. **(E)** Immunohistochemical images of CD4^+^ and CD8^+^ T cells in tumor tissues from each group and quantitative analysis of the proportion of positive cells. (Data are shown as the mean ± SEM. *P < 0.05, **P < 0.01, ***P < 0.001, ****P < 0.0001; ns, no significance).

To investigate the effect of arsenic sulfide on the HCC response to PD - 1 antibodies *in vivo*, we used Hepa1–6 HCC cells to establish subcutaneous implantation model in C57BL/6 mice. As shown in the treatment schedule (**Figure 6A**), mice were randomly divided into 4 groups and treated with Vehicle+IgG, Anti-PD-1, arsenic sulfide, or the combination with Anti-PD-1 and arsenic sulfide until the study endpoint. The tumor volumes of the four groups at baseline did not differ before therapy (**Supplementary Figure S4A**). The combination therapy resulted in a more significant reduction in tumor size and growth compared to the control group (P < 0.001), Anti-PD-1 alone (P < 0.05), and arsenic sulfide alone (P < 0.001) (**Figure 6B**, **Supplementary Figure S4B**). To further explore the underlying mechanisms of the synergistic effect of arsenic sulfide and Anti-PD-1, we tested the immune cell profiles in the peripheral blood and tumor-infiltrating lymphocytes. In peripheral blood, the combination therapy enriched CD8+ T cells, with their proportion increasing from 6.17% in the control group to 18.05% in the combined treatment group (**Figure 6C**). Furthermore, arsenic sulfide treatment significantly reduced PD - 1 expression on the surface of CD8^+^ cells (**Figure 6C**). Additionally, CD8^+^ T cells and CD4^+^ T cells were significantly increased in tumor-infiltrating lymphocytes (**Figure 6D**). Consistent with the above results, immunohistochemistry (IHC) staining also showed an increase in CD8^+^ T cells and CD4^+^ T cells in Hepa1–6 tumors after the combination with Anti-PD-1 and arsenic sulfide treatment (**Figure 6E**). Analysis of tumor lysates from Vehicle +IgG (M1, M2) and arsenic sulfide-treated (M3, M4) mice showed an obvious decrease in the expression of THBS1 proteins (**Supplementary Figure S4C**). Collectively, these data support that the combination of PD - 1 blockade with arsenic sulfide significantly enhances their immune response to HCC cells, leading to greater antitumor efficacy.

## Discussion

**Figure 7:**
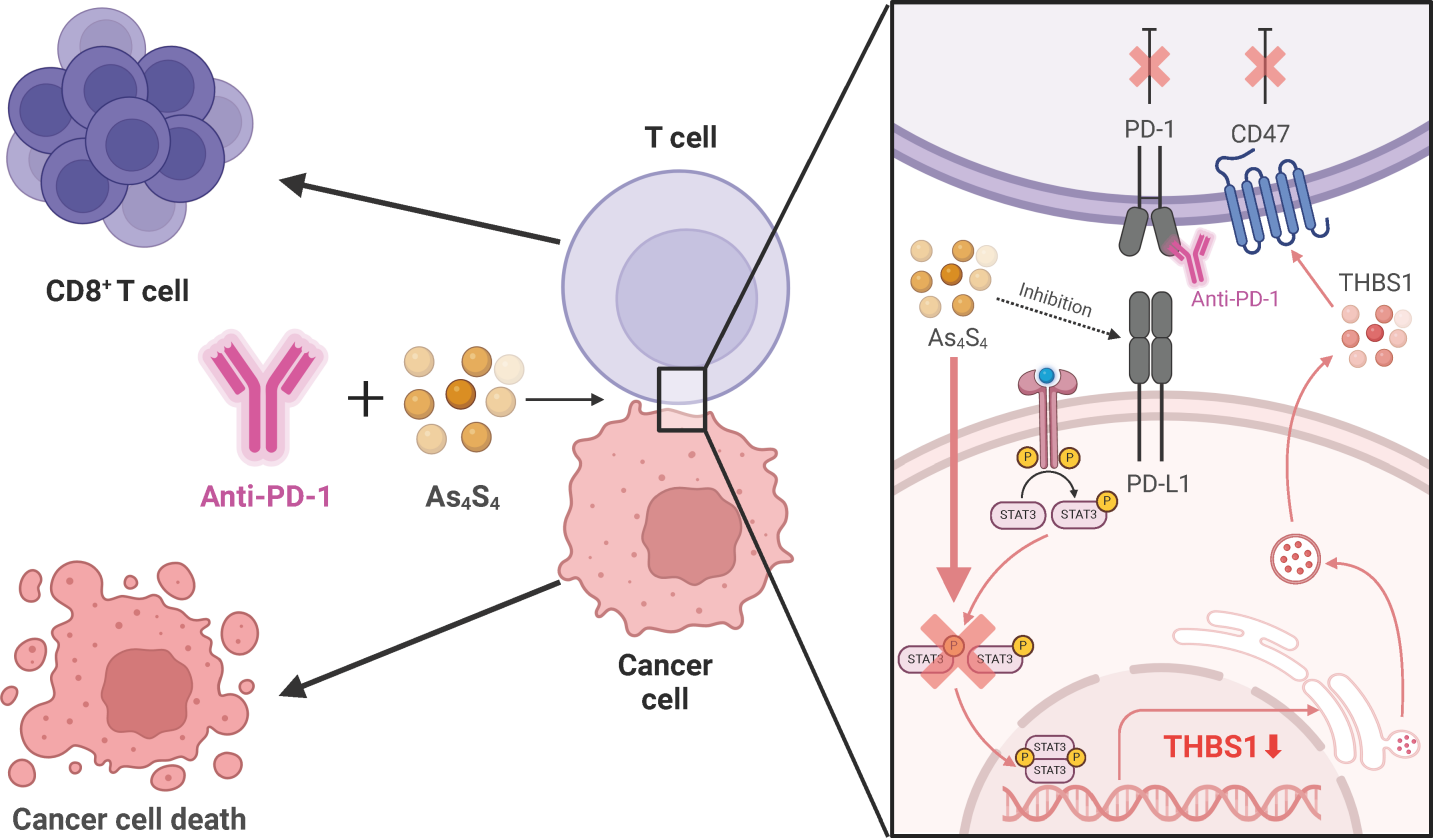
Schematic Representation of How Arsenic Sulfide Enhances the Therapeutic Effect of Hepatocellular Carcinoma Immunotherapy through the STAT3-THBS1/CD47 Pathway. Arsenic sulfide has the ability to attract cytotoxic CD8^+^ T cells into the HCC microenvironment and reduce the expression of THBS1 through the STAT3-THBS1 signaling pathway. This reduction disrupts the interaction between THBS1 and CD47, which in turn increases the activation of T cells. The combination of PD - 1 blockade with arsenic sulfide treatment can further enhance the cytotoxic capabilities of CD8^+^ T cells, resulting in a significantly synergistic therapeutic effect against HCC.

Over the last ten years, there has been considerable progress in the immunotherapy that targets immune checkpoints of advanced HCC. However, despite the introduction of innovative treatment approaches, they have not been universally effective. Regrettably, the failure of response to mono-agent therapy or relapses after ICIs alone remains frequent. Here, we report that combination therapy comprising arsenic sulfide administration and PD - 1 blockade resulted in significant tumor repression in murine HCC models. We found that arsenic sulfide has the ability to attract cytotoxic CD8^+^ T cells into the HCC microenvironment and reduce the expression of THBS1 in tumor through the STAT3-THBS1 signaling pathway. Sequentially, this reduction disrupts the interaction between THBS1 and CD47, which in turn increases the activation of T cells. The combination of PD - 1 blockade with arsenic sulfide treatment can further enhance the cytotoxic capabilities of CD8^+^ T cells, resulting in a significantly synergistic therapeutic effect against HCC (**Figure 7**).

Recent studies have reported that certain conventional reagents, such as IFNα, which is commonly used in clinical practice, can synergize with PD - 1 blockade to enhance the immune checkpoint blockade response in HCC (32, 33). Repurposing existing drugs for novel therapeutic applications offers a significant advantage: it expedites the clinical application process, bypassing the lengthy and costly stages of new drug development. Arsenic sulfide has been a mainstay in the treatment of acute promyelocytic leukemia for more than two decades. Our research group, along with others, has discovered that it possesses the ability to inhibit solid tumors, particularly those of the digestive system (14, 18). Notably, as a single agent, arsenic compounds did not benefit patients diagnosed with solid tumors (34). However, when it was combined with other agents, treatment benefit emerged (34). Our *in vitro* and *in vivo* experiments demonstrate that the combination of arsenic sulfide with PD - 1 antibodies results in a synergistic effect on HCC. The enhancement of T cell activation in the presence of arsenic sulfide, indicate an immunostimulatory effect that complements the immune checkpoint blockade. As we all know, higher CD8^+^ T cell IHC scores correlate with a more favorable response to ICIs in HCC (35, 36). The *in vivo* data, showing increased CD8^+^ T cell infiltration and reduced PD - 1 expression on these cells, further support the notion of a robust immune response triggered by the combination therapy.

In chronic infections and tumors, persistent antigen stimulation induces T cell exhaustion (37). Exhausted CD8+ T cells (Tex) exhibit a distinct transcriptional and epigenetic state driven by TOX and express inhibitory receptors like PD - 1, LAG - 3, CTLA - 4, and TIM3 (38, 39). Recently, a phase III clinical trial called RELATIVITY - 047 demonstrated that the combination of LAG - 3 antibody and PD - 1 antibody has better clinical anti-tumor effects (40, 41). Mechanistically, blockade of both LAG - 3 and PD - 1 modulated CD8^+^ T cell differentiation, leading to co-expression of cytotoxic and exhaustion gene modules (42). PD - 1- and LAG - 3-deficient CD8^+^T cells were transcriptionally distinct, with broad TCR clonality and enrichment of effector-like and interferon-responsive genes, resulting in enhanced IFN-γ release indicative of functionality (43). We have shown here that arsenic sulfide treatment significantly reduced the expression of exhaustion marker LAG3. Co-culturing T cells with tumor cells induced LAG3 expression in 30% of T cells, a percentage that significantly dropped to 21% and 10% with the addition of 0.5 µM and 1 µM arsenic sulfide, respectively. Moreover, arsenic sulfide strikingly increased IFN-γ expression, indicating a potential enhancement of anti-tumor immune responses. These observations provide a mechanistic explanation for the synergistic effects of targeting PD - 1 and arsenic sulfide where loss of PD - 1 provides a strong numerical expansion and arsenic sulfide fosters enhanced effector biology, including improved cytotoxicity.

It has been recently reported that a positive link between high THBS1 expression and mesenchymal characteristics, immunosuppression, and unfavorable colorectal cancer prognosis (20). The suppression of THBS1 expression effectively curbs tumor cell invasion and proliferation in glioblastoma (44). Our genomic analysis identified THBS1 as a differentially expressed gene in HCC, a finding that aligns with its emerging role as a prognostic factor in Asian populations. The downregulation of THBS1 by arsenic sulfide suggests a potential mechanistic link between gene expression and therapeutic response. The transcriptional regulation of THBS1 by p-STAT3, further modulated by arsenic sulfide, underscores the intricate network of molecular interactions that govern HCC progression and response to therapy. Our results highlight the potential of targeting the p-STAT3-THBS1 axis as a therapeutic strategy, a concept supported by the significant reduction in THBS1 expression following treatment with STAT3 inhibitors.

THBS1 exerts different effects by binding to different cellular receptors (19). The binding between VSIG4 and THBS1 protein facilitating the malignant progression of glioma cells (45). The binding of THBS1 to CD47 controlled adaptive immunity via inhibiting T-cell activation and differentiation (46). Specific inhibition of the THBS1/CD47 interaction using an antagonist peptide decreases cell invasion in glioblastoma (44). The immunomodulatory role of THBS1 in HCC is further elucidated by our findings that THBS1 inhibits T cell-mediated cytotoxicity, potentially through the CD47 pathway. This discovery is pivotal, as it provides a molecular explanation for the observed immune evasion in HCC. The ability of arsenic sulfide to counteract THBS1’s suppressive effect on T cells, thereby enhancing immune cell-mediated killing, is a significant advancement. This finding suggests that arsenic sulfide may not only exert direct cytotoxic effects on HCC cells but also modulate the tumor microenvironment to favor immune cell infiltration and activity. The proposed model, wherein arsenic sulfide disrupts the THBS1-CD47 interaction, leading to enhanced T cell activation and cytotoxicity, provides a compelling framework for understanding the observed therapeutic effects. The disruption of this interaction by arsenic sulfide, in conjunction with PD - 1 blockade, may represent a novel approach to enhance the immune response against HCC. This strategy could potentially be extended to other cancers where immune evasion is a critical barrier to effective therapy.

Although our study provides important insights into the role of THBS1 and the potential of arsenic sulfide combined with PD - 1 blockade in HCC, several limitations should be acknowledged. First, the scope of this study was limited to a few *in vitro* cell lines and animal models. Given the high heterogeneity of HCC, further validation in additional cell lines and human clinical trials is warranted. Moreover, the molecular mechanisms underlying the synergistic effects of arsenic sulfide and PD - 1 antibodies require deeper investigation, particularly in the context of interpatient variability.

Overall, our findings suggest a promising therapeutic avenue for HCC, highlighting the potential of arsenic sulfide combined with PD - 1 inhibitors to enhance immune-mediated tumor clearance. Future studies should focus on translating these preclinical findings into clinical applications, with particular emphasis on patient stratification and personalized treatment strategies to support novel combination approaches for HCC immunotherapy.

## Data Availability

Publicly available datasets were analyzed in this study. This data can be found here: GEO (GSE87630) and ImmPort database.
