# Supplementary material for: Arsenic sulfide enhances the therapeutic effect of hepatocellular carcinoma immunotherapy through STAT3-THBS1/CD47 pathway
Source: Front Immunol. 2025 Sep 11;16:1612318. doi: 10.3389/fimmu.2025.1612318 (PMC12460339; doi:10.3389/fimmu.2025.1612318)
Supplement: Supplementary file 1 [file Table1.docx]

**Supplementary Information**

Arsenic sulfide enhances the therapeutic effect of hepatocellular carcinoma immunotherapy through STAT3-THBS1/CD47 pathway

Ting Kang^1#^| Zhuowei Feng^1^^#^| Yu Cai^1^| Ruizhe Huang^1^| Ruiheng Wang^2^| Zhiyi Liu^1^| Shumin Lu^1^| Shufeng Xie^2^| Han Liu^2^| Siyu Chen^1*^

1.Department of Oncology, Xin Hua Hospital, School of Medicine, Shanghai Jiao Tong University, Shanghai 200092, China

2.Shanghai Institute of Hematology, State Key Laboratory of Medical Genomics, National Research Center for Translational Medicine at Shanghai, Ruijin Hospital, Shanghai Jiao Tong University School of Medicine and School of Life Sciences and Biotechnology, Shanghai, China.

# Ting Kang and Zhuowei Feng Contributed equally to this work.

* **Correspondence:**

**Siyu Chen**, Department of Oncology, Xin Hua Hospital, School of Medicine, Shanghai Jiao Tong University, Shanghai 200092, China. Tel: +86-21-25077642; Phone +86 13651687212;

E-mail: siyu.chen@shsmu.edu.cn.

**This file includes:**

Supplementary Figures S1-S4.

Supplementary Table 1

**Supplementary figures**

**Figure.S1** **Arsenic sulfide inhibits viability of HCC, promotes apoptosis, and suppresses the expression of THBS1.** (A)Cell proliferation assays showing the effect of arsenic sulfide on the growth of Hep3B. (B) Immunoblot analysis of THBS1 expression in arsenic sulfide treated Hep3B cell. (C) Immunoblot analysis of THBS1 expression in different time of 3μM arsenic sulfide treated HepG2 and Hep3B cells. (D) Flow cytometry analysis quantifying apoptosis in Hep3B cells exposed to different concentrations of arsenic sulfide. (E) and (F) Flow cytometry analysis quantifying apoptosis in HepG2 (E) and Hepa1-6 (F) cells exposed to different time of 3μM arsenic sulfide. (Data are shown as the mean ± SEM, *n*=3. **P* < 0.05, ***P* < 0.01, ****P* < 0.001; ns, no significance).

**
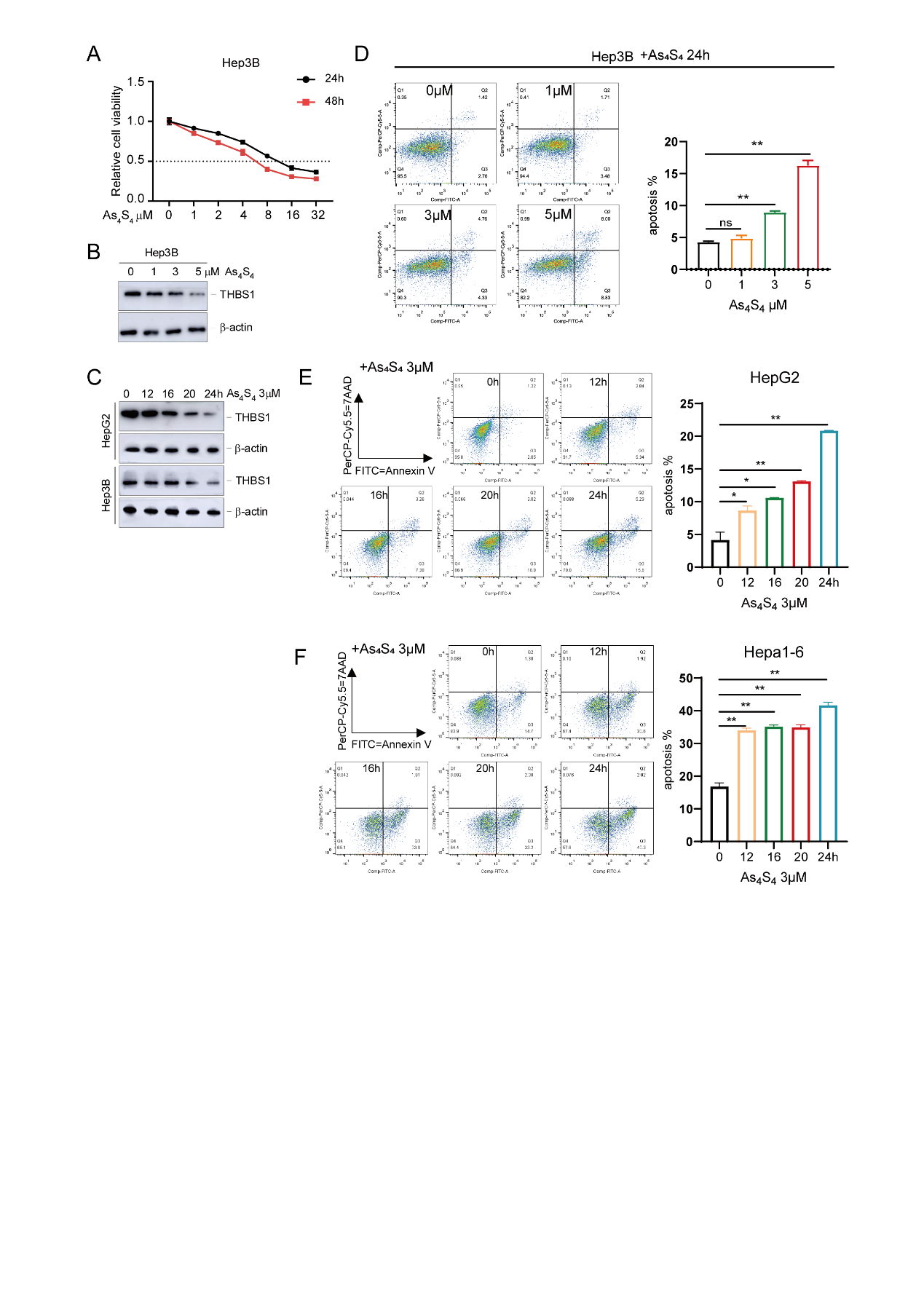
**

**Figure.S2** **Arsenic sulfide suppresses THBS1 expression by inhibiting STAT3 phosphorylation and transcriptional activity.**

(A) Immunoblot analysis of p-STAT3 and STAT3 expression in arsenic sulfide treated HepG2 and Hepa1-6 cells. (B) Predicted binding sites of STAT3 on the THBS1 promoter.

(swissregulon.unibas.ch/sr/). (C) Median STAT3-THBS1 pathway activation scores in different Immunophenoscore (IPS) groups for PD-1, CTLA-4, and combined immune checkpoint inhibitors. (D) Kaplan-Meier survival analysis based on STAT3-THBS1 pathway activation scores in HCC patients. (E) Spearman correlation analysis between STAT3-THBS1 pathway activation scores and the expression levels of immune checkpoint-related genes. (F) IGV visualization of STAT3 ChIP-seq signal tracks across the THBS1 genomic region.

**
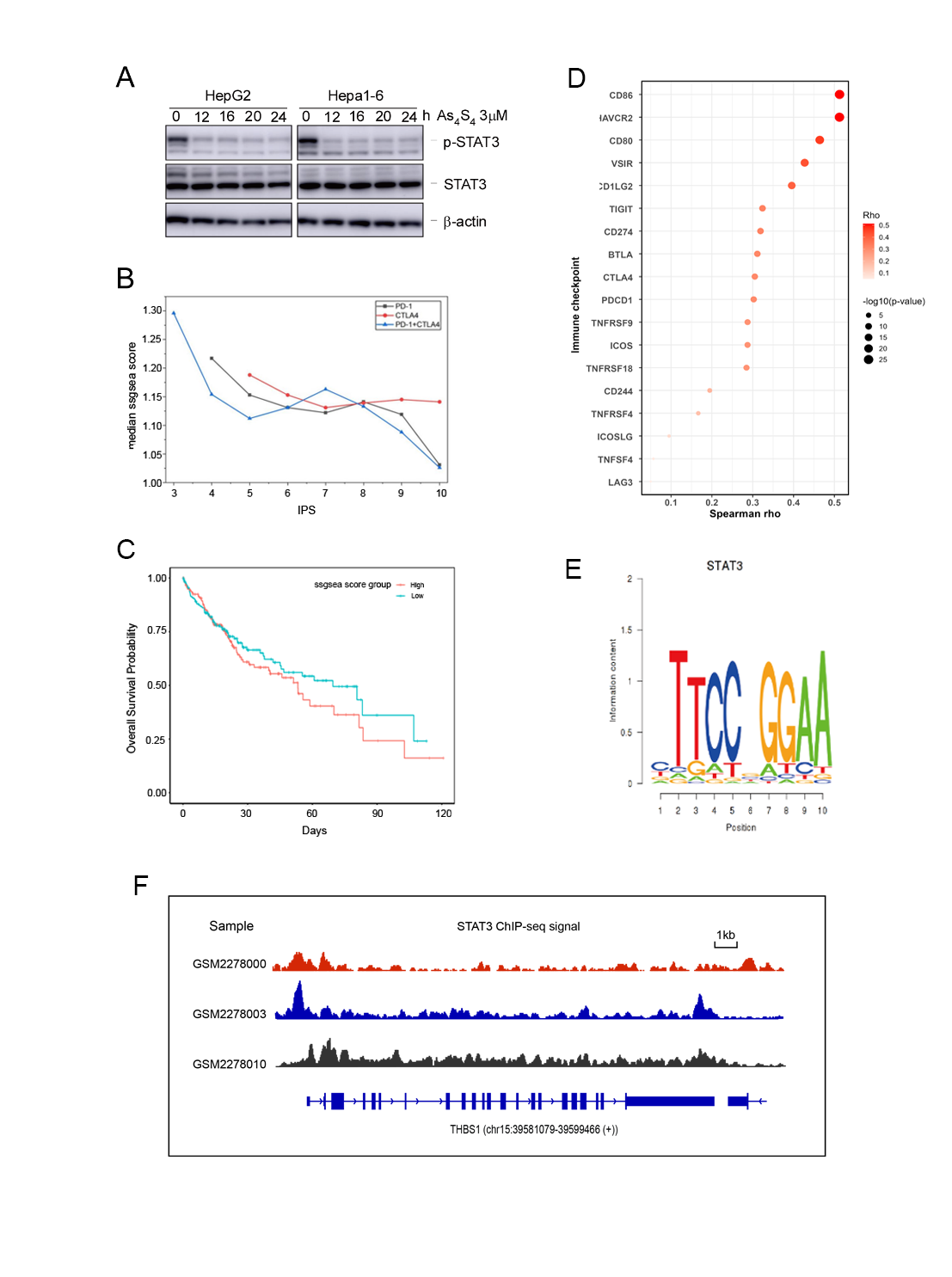
**

**Figure.S3 Enhanced Therapeutic Efficacy of Combination Therapy with PD-1 Blockade and Arsenic Sulfide *In Vitro***

(A) Flow cytometry analysis assessing T cell activation by measuring the expression of CD69. (B) Flow cytometry analysis of multiplex cytokine profiling to detect secretion of cytokines (IL-2, IL-6, IL-10, Perforin, sFasL, sFas, IL-17A, Granzyme A, Granzyme B, Granulysin, IL-4) by T cells after treated with different drugs.


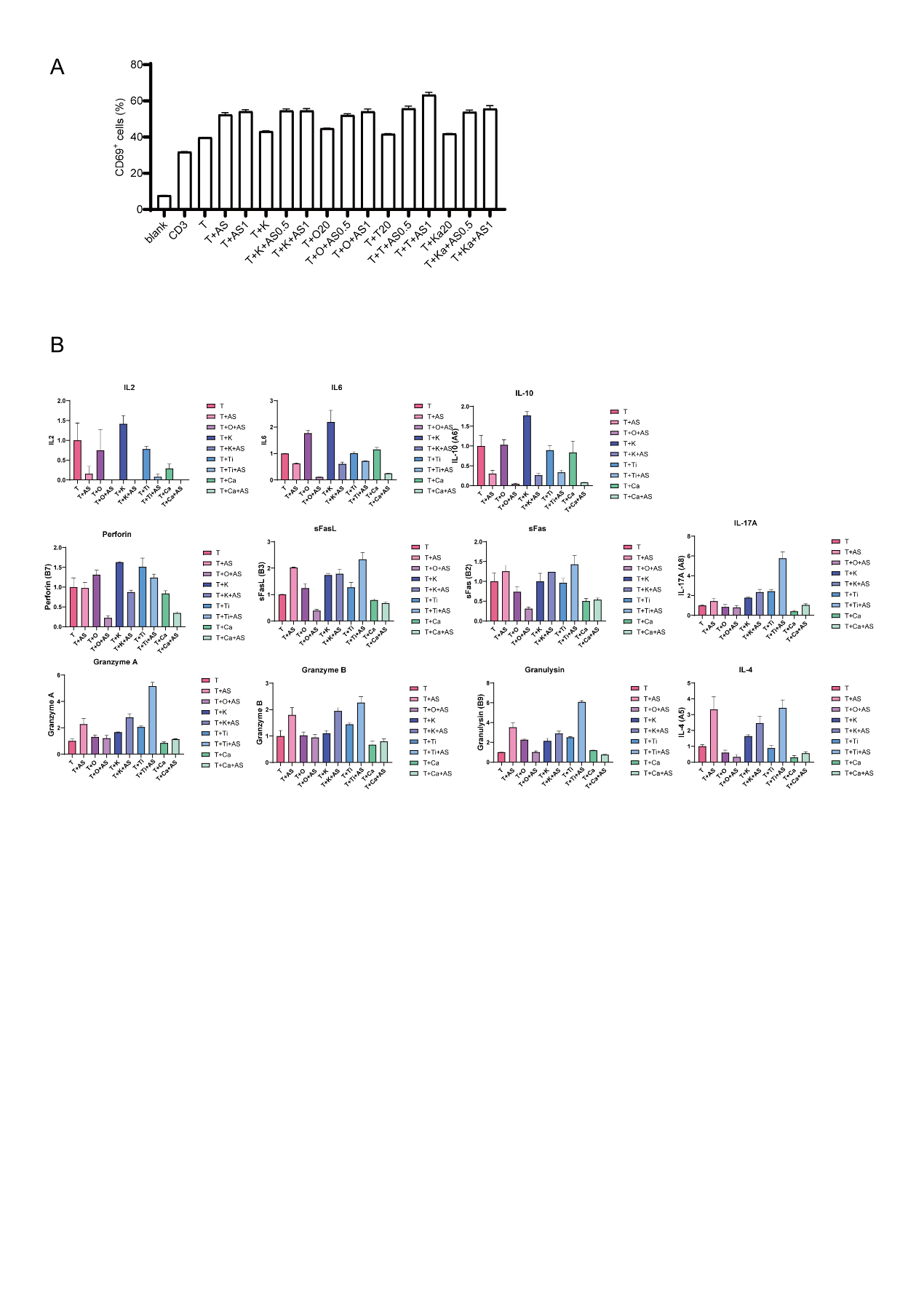


**Figure.S4 Arsenic Sulfide Enhances the Efficacy of PD-1 Blockade Against HCC In Vivo**

1. and (B) Tumor volume of Random(A) and Sacrifice(B). (C) Immunoblot analysis of indicated protein expression in excised tumors. Fold changes relative to first line are indicated. (Data are shown as the mean ± SEM. **P* < 0.05, ***P* < 0.01, ****P* < 0.001; ns, no significance).


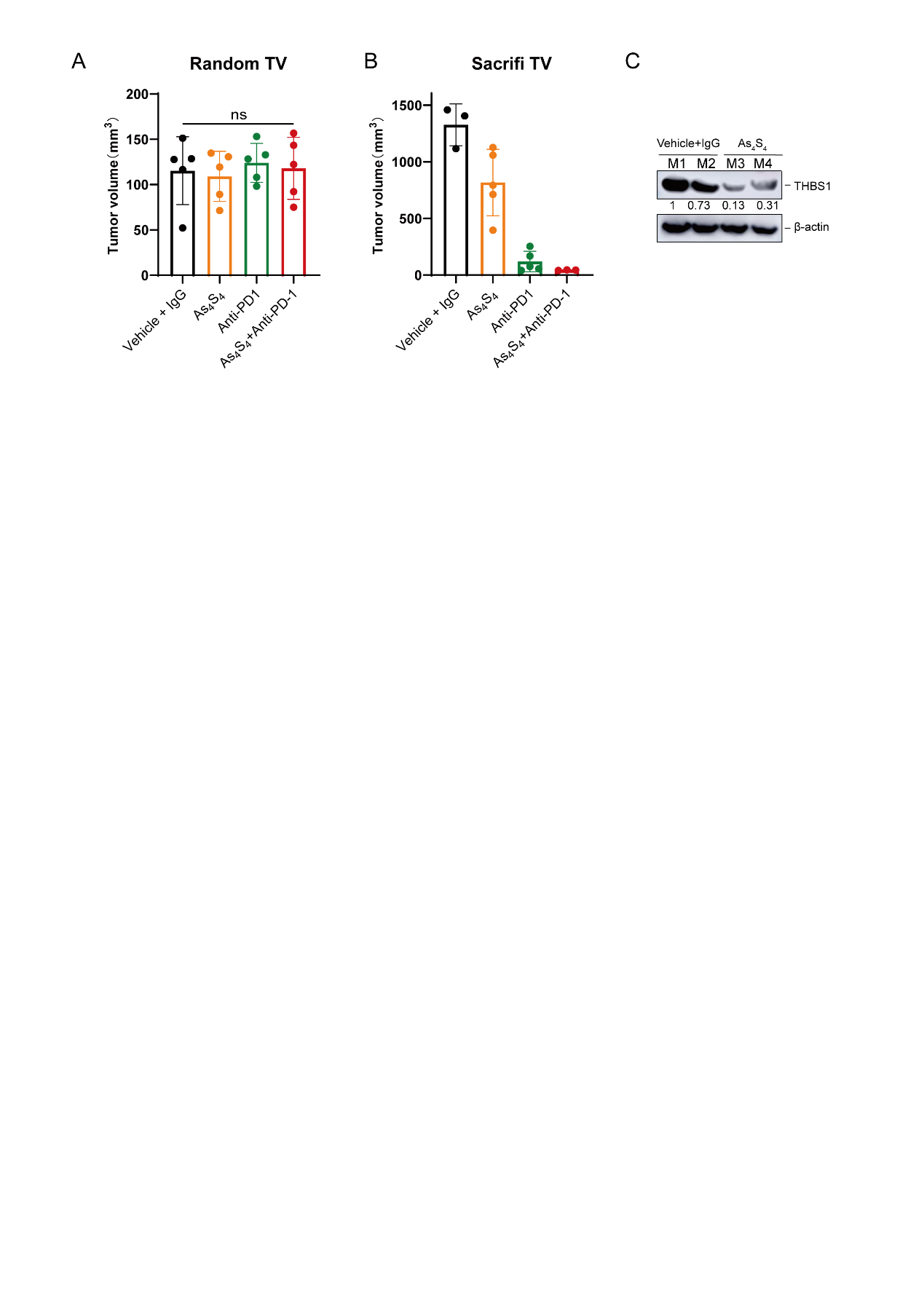


**Supplementary Table**

**Supplementary Table 1. Primer sequences for Real-time PCR.**

| primer | sequence |
| --- | --- |
| HUMAN-THBS1-qPCR-F | TGCTATCACAACGGAGTTCAGT |
| HUMAN-THBS1-qPCR-R | GCAGGACACCTTTTTGCAGATG |
| HUMAN-GAPDH-qPCR-F | ggcacagtcaaggctgagaatg |
| HUMAN-GAPDH-qPCR-R | atggtggtgaagacgccagta |
| MOUSE-THBS1-qPCR-F | TTCTTACCCTTGACAACAACGTG |
| MOUSE-THBS1-qPCR-R | CCACAGATAGCTTGGAGGTCC |
| MOUSE-GAPDH-qPCR-F | AGGTCGGTGTGAACGGATTTG |
| MOUSE-GAPDH-qPCR-R | TGTAGACCATGTAGTTGAGGTCA |
| HUMAN-THBS1-ChIP-PCR-F | atgcttgctgatcaccc |
| HUMAN-THBS1-ChIP-PCR-R | cgcaactttccagctaga |
| MOUSE-THBS1-ChIP-PCR-F | TGGCTTCCTCTGTGGTCTCT |
| MOUSE-THBS1-ChIP-PCR-R | GTCAAGGTCATGGGATGGTC |
